# Supplementary material for: Fc-Independent Protection from SARS-CoV-2 Infection by Recombinant Human Monoclonal Antibodies
Source: Antibodies (Basel). 2021 Nov 8;10(4):45. doi: 10.3390/antib10040045 (PMC8628512; doi:10.3390/antib10040045)
Supplement: Supplementary file 1 [file antibodies-10-00045-s001.zip › antibodies-1404001-supplementary.pdf]

Supplementary Data

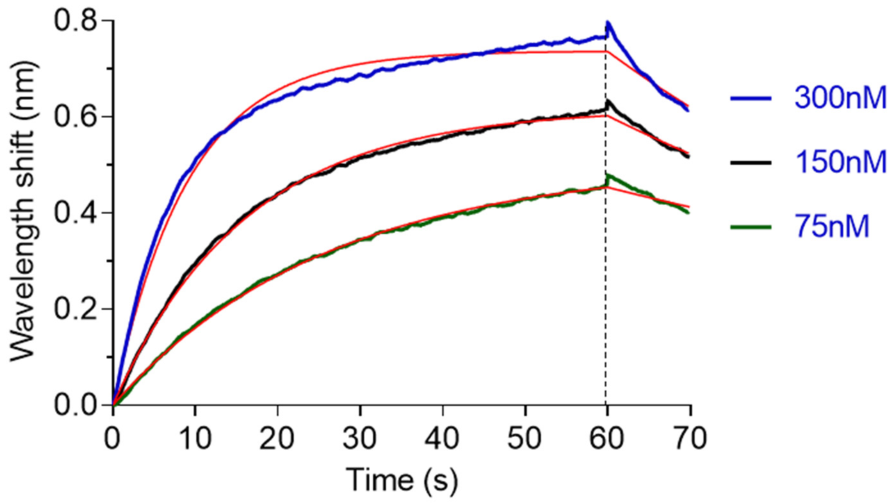

Supplementary Figure S1. Binding of MD65-AG to hFcRn. Anti-Fab coated sensors were loaded with MD65-AG (30 µg/ml) to reach 2.5 nm wavelength shift, and then washed. Sensors were then incubated with the indicated concentrations of hFcRn in pH 6.0, for 60 sec (association phase) and transferred to buffer-containing wells for additional 10 sec (dissociation phase). Binding and dissociation were measured as changes over time in light interference after subtraction of parallel measurements from unloaded biosensors. Sensorgrams were fitted (Red lines) with a 1:1 binding model.
